# The Role of Mental Health Conditions in the Diagnosis of Neurocognitive Impairment in People Living with HIV

**DOI:** 10.3390/diagnostics10080543

**Published:** 2020-07-30

**Authors:** Irene Portilla-Tamarit, Nicolás Ruiz-Robledillo, Marcos Díez-Martínez, Rosario Ferrer-Cascales, Cristian Alcocer-Bruno, Joaquín Portilla

**Affiliations:** 1 Department of Health Psychology, University of Alicante, 03690 Alicante, Spain; irene.portilla@ua.es (I.P.-T.); rosario.ferrer@ua.es (R.F.-C.); cristian.albru@ua.es (C.A.-B.); 2 Alicante Institute for Health and Biomedical Research (ISABIAL–FISABIO Foundation), 03010 Alicante, Spain; diez_marmar@gva.es (M.D.-M.); joaportilla@gva.es (J.P.); 3 Department of Infectious Diseases, General University Hospital of Alicante, 03010 Alicante, Spain; 4 Spanish Network of Excellence on HIV Research, RIS, 28029 Madrid, Spain; 5 Department of Clinical Medicine, Miguel Hernández University, 03016 Alicante, Spain

**Keywords:** mental health conditions, neurocognitive impairment, HIV infection, HIV-suppressed viremia, behavior disorders

## Abstract

The aims of the present study were to evaluate the prevalence of undiagnosed mental health conditions (UMHC) in people living with HIV (PLWHIV) on antiretroviral treatment and with long-term suppressed HIV viremia, and its association with neurocognitive impairment (NCI). A cross-sectional observational study on HIV subjects, ≥18 years old, on stable antiretroviral treatment and with HIV viral load <50 copies/mL was carried out. Patients with known comorbidities, substances abuse, anxiety or depression were excluded. UMHC were evaluated by the Millon Clinical Multiaxial Inventory-III and NCI by Frascati criteria. The association between NCI and sociodemographic, clinical HIV variables and mental health conditions was analyzed. Further, the relationship between mental health conditions scores and NCI diagnosis was evaluated. Eighty patients were included, 37.5% had at least one undiagnosed mental health condition, and 26.3% had NCI. The most frequent mental health conditions were: anxiety (21.3%); bipolar disorder (11.3%); and substance dependence (8.8%). Only longer time since HIV diagnosis (*p* = 0.030) and at least one mental health condition diagnosis (*p* = 0.002) showed an association with NCI. Participants with NCI presented higher scores in anxiety, alcohol dependence and post-traumatic stress. Undiagnosed mental health conditions are frequent in PLWHIV. These disorders cannot be identified by HIV clinicians or basic screening questionnaires, and they are not usually self-reported by patients. UMHC could act as confounders in the evaluation of NCI.

## 1. Introduction

Neurocognitive impairment (NCI) without an alternative explanation than HIV infection has been described in people living with HIV (PLWHIV) [1]. Diagnosis of NCI in PLWHIV is based on standardized neuropsychological tests and the exclusion of health problems [2]. Based on established criteria and the severity of symptoms, NCI is categorized as asymptomatic neurocognitive impairment (ANI), minor neurocognitive disorder (MND) or HIV-associated dementia [2]. This non-AIDS event has severe negative consequences, such as poor adherence to antiretroviral therapy (ART) [3], difficulties in performing activities of daily living [4], loss of employment [5], a reduction in quality of life [6] and higher mortality risk [7], among others.

There is no consensus about the prevalence of NCI in PLWHIV [8,9,10,11,12,13,14,15]. These discrepancies between studies could be related to several factors. In this regard, the most important variables identified in the previous research are based on the heterogeneous population included in the studies, differences in HIV infection control or the lack of the identification of several comorbidities, something that could increase the prevalence of NCI, such as mental health conditions [10,12,13].

Mental health conditions are more common in PLWHIV (63%) as compared to the HIV-negative population (30.5%) [16]. Hence, when compared to their HIV-negative same sex counterparts, HIV-positive men were more likely to have any mood disorder (odds ratio (OR) = 6.10), major depressive disorder/dysthymia (OR = 3.77), any anxiety disorder (OR = 4.02) and any personality disorder (OR = 2.50) [17]. The mechanisms underlying this higher prevalence in PLWHIV could be related to the high exposition of this population to multiple stressful situations, such as stigmatization [18], decreased social support and concerns after the HIV diagnosis [19].

Although both NCI and mental health conditions are highly prevalent in this population, their bidirectional influence remains unclear, although it can influence the diagnosis of both. International guidelines that have specific sections regarding diagnosis of HIV-associated NCI recommend a complete assessment including a comprehensive clinical history and examination, screening for depression, neuropsychological testing, cerebral MRI scanning and lumbar puncture [20]. For instance, European AIDS Clinical Society (EACS) guidelines recommend to exclude severe psychiatric conditions, abuse of psychotropic drugs, alcohol abuse and depression, in order to confirm that NCI is caused directly by HIV infection and not by a cofounder factor [21]. Despite these recommendations, most of studies on NCI prevalence usually evaluate only depression and anxiety [14,22], insomnia [23] or alcohol abuse [24]. The majority of studies do not evaluate and identify the presence of other mental health conditions that could also have an influence on cognitive performance [25]. Moreover, most of these studies evaluate only depression and anxiety through the employment of screening scales or questionnaires, originally designed for the identification of symptomatology and not mental health conditions [23,25]. Moreover, some studies do not follow these recommendations and do not evaluate any mental health condition in this regard [26,27].

Furthermore, other facts related to these issues are based on the employment of screening tools for mental health conditions which present some limitations, fundamentally, the lack of an exhaustive and wide-range evaluation of mental health conditions beyond anxiety and depression symptoms [28]. This could explain why some studies find a relationship between mental health conditions and NCI [29], while others do not [30].

As has been previously reported, mental health conditions and NCI can coexist in PLWHIV [31]. The Mind Exchange Working Group has reported that mental health conditions in HIV people can act as a cofounder in NCI diagnosis [32]. For that reason, authors recommend to evaluate alcohol or drug dependence, depression, anxiety and post-traumatic stress disorder, among others, before NCI diagnosis [32]. Therefore, the lack of an exhaustive evaluation of mental health conditions in PLWIH, with accurate and adequate evaluation instruments, could be on the basis of the discrepancies in the influence of mental health conditions on NCI. In this regard, these controversial results make it necessary to carry out new studies in this population to clarify this relationship, emphasizing the necessity of employing previously validated diagnostic tests, with adequate psychometric properties, for the evaluation of mental health conditions in-depth.

In a previous study of our group, the prevalence of NCI in “healthy” PLWHIV, with good adherence to ART and long-term suppressed viremia and without medical or psychiatric comorbidities, illegal drugs or alcohol abuse or psychotropic drugs users, was analyzed. The presence of depression or anxiety was identified by the Hospital Anxiety and Depression Scale (HADS) questionnaire in all patients. It was observed an NCI prevalence of 29.8% (95% CI: 21.0–40.2). Time since HIV diagnosis and increasing levels of interleukin-6 were the only factors associated with NCI diagnosis [11]. Going one step further, the aims of the present study were to evaluate the prevalence of undiagnosed mental health conditions (UMHC) in the same population and to analyze the possible association between mental health conditions and NCI diagnosis.

## 2. Materials and Methods

### 2.1. Study Design and Participants

The study is a cross-sectional observational study. The sample of the present study was composed of 80 PLWHIV. The inclusion criteria to participate in the study were: HIV infection, ≥18 years-old, unchanged ART for at least one year, HIV-VL <50 copies/mL over the previous 12 months and completed HADS [33] questionnaire with a score less than 10 points. The exclusion criteria were: self-reported poor adherence to ART (<95% of antiretroviral drugs doses during previous month), smoking or substance abuse (alcohol, methadone, psychotropic and recreational drugs) self-reported by patients or described in the electronic medical reports during last 12 months, traumatic brain injury in the last 12 months and presence of comorbidities identified in medical records: dementia or other central nervous system diseases, mental health conditions diagnostic, viral chronic hepatitis, active cancer or infection, diabetes mellitus, high blood pressure, cardiovascular disease, hypothyroidism or malnutrition. Finally, mental or physical impairment that could hinder participants’ ability to complete or understand the study questionnaires.

### 2.2. Variables

Sociodemographic and clinical HIV variables. Demographic variables were sex, age and education level. HIV-related variables included HIV transmission mechanism, time since HIV diagnosis, current and nadir CD4+ lymphocyte count.

Neurocognitive disorder (NCI). Frascati criteria were used for the diagnosis of neurocognitive impairment. Based on the results of the neurocognitive evaluation, patients were classified as ANI, MND or HIV-associated dementia [2]. ANI was defined by scores below 1.0 standard deviation (SD) on the demographically corrected mean at least in 2 neuropsychological domains with no interference in daily functioning. MND was categorized with same scores of ANI but in this case with interference in daily functioning. HIV-associated dementia was defined by at least two domains with a score of ≥2 SDs below the mean, with significant interference in daily function.

To asses interference in daily life, patients were asked if cognitive impairment interfered or not, at least mildly, in one of the following areas: work, household, mental acuity, tasks or social activities [2]. This variable was expressed as a dichotomous categorical variable (yes/no).

Mental health conditions. For the assessment of mental health conditions, the Spanish version of the Millon Clinical Multiaxial Inventory-III (MCMI-III) was employed [34,35]. This is a 175-item self-report scale with two response options (true or false) that assesses 14 personality disorders and 10 clinical syndromes. For the present study, only mental health conditions were evaluated: anxiety disorder, somatoform disorder, bipolar disorder, dysthymic disorder, alcohol dependence, substance dependence, post-traumatic stress, thought disorder, major depression and delusional disorder. The MCMI-III was corrected online on the platform of Pearson Clinical & Talent Assessment, Q-global. MCMI-III has good psychometric properties, with a test–retest reliability between 0.84 and 0.96 and an internal consistency higher than 0.80. Regarding the internal consistency of each scale, the validation of the questionnaire exhibited an adequate reliability index, exceeding in more than 21 scales the value of the coefficients alpha 0.75. Results are expressed according to the MCMI-III criteria, where raw scores are transformed into prevalence scores. Following the authors’ instructions, a score >75 points was employed as a cut-off score in each item to define the presence of mental health conditions, being classified as “presence” or “absence”. Finally, patients were categorized as having “at least one mental health conditions” or not [35].

#### Procedure

In the first meeting, after informed consent was signed by participants, a clinician and a neuropsychologist reviewed all electronic clinical reports shared with psychiatrists and family doctors and performed a structured clinical interview to ensure the fidelity of inclusion/exclusion criteria prior to the administration of neuropsychological tests. Further, participants self-completed an anxiety and depression screening questionnaire (HADS) [33]. If participants scored 10 or more, they were excluded from participation in the study. The sociodemographic and clinical variables were collected from the medical records.

In the second visit, a trained neuropsychologist conducted a personal interview with each participant, lasting approximately 120 min and consisting of an administration of a comprehensive neurocognitive battery that covers seven neurocognitive domains (ND), following the established criteria by international consensus [2]. This battery was composed by the following two tests to evaluate each ND: speed of information processing (Trail making Test Form A and WAIS-IV Digit Symbol); verbal fluency (PMR and animals); attention/working memory (WAIS-IV Digit Span and D2, Test of Attention); executive function (Trail making Test Form A, Five Digit Test and The Towel of London Test); motor function (Grooved Pegboard Test and The MacQuarrie Test); learning (TAVEC—Consistent Long-Term Retrieval and Brief Visuospatial Memory Test-Revised-Total Learning) and delayed recall (TAVEC-delayed recall and Brief Visuospatial Memory Test-delayed recall). Spanish versions of all the tests were used. *Z*-scores were obtained of raw corrected by age, education level and sex from Spanish normative data. The neuropsychology battery was administered always by the same neuropsychologist.

In a third visit, the same neuropsychologist conducted a personal interview with each participant, lasting approximately 30 min and consisting of an administration of the Millon Clinical Multiaxial Inventory-III (MCMI-III) [35].

The study was carried out in the Infectious Diseases Unit of the General University Hospital of Alicante in Spain, which attends approximately 1250 PLWHIV. All patients in usual care between November 2014 and February 2015 who met the inclusion criteria were invited to participate in the study. Only 8 patients, who complied with the inclusion criteria, refused to participate in the study, primarily due to a lack of time to perform the neuropsychological tests. Four patients were also excluded because they did not complete properly the Millon Clinical Multiaxial Inventory-III.

The study was approved (26 November 2014) by the Ethics Committee of the General University Hospital of Alicante (PI2013/25). The project was carried out in accordance with good clinical practice standards and international ethical principles applicable to medical research in humans. All participants provided written informed consent.

### 2.3. Data Analysis

To describe the population in qualitative variables, absolute frequencies and percentages were used. With regard to quantitative variables, the Kolmogorov–Smirnov test was employed to determine the normality of the data distribution. Further, quantitative variables were dichotomized using clinical criteria (time since HIV diagnosis) or variable distribution (age). The prevalence of NCI and mental health conditions was calculated with a 95% confidence interval (CI). Chi-squared test was employed to assess associations between NCI sociodemographic, clinical HIV variables and mental health conditions. Further, the crude odds ratio (OR) and 95% CI were calculated. Variables which showed a significant (*p* < 0.05) association in bivariate analysis were fitted in a logistic regression model to calculate the adjusted OR. Moreover, each mental health conditions was analyzed as a continuous variable to study the relationship between their scores and the NCI diagnosis by the student´s *t*-test or Mann–Whitney *U* test as appropriate. The level of statistical significance in the hypothesis testing was *p* < 0.05. For data analysis, the software package IBM-SPSS Statistics 25.0 (Armonk, NY, USA) was used.

## 3. Results

### 3.1. Sociodemographic and Clinical Characteristics of the Sample

Eighty patients were included, 77.5% were males with a mean age of 46.1 ± 7.6 years, and 66.3% had secondary or university studies. Concerning clinical variables, the mean of time since diagnosis was 12.4 ± 7.1 years (Table 1).

### 3.2. Prevalence of NCI and Mental Health Conditions

The NCI prevalence observed was 26.3% (16.1–36.5). Using Frascati criteria, patients were categorized as ANI: 13 (16.3%); MNI: 7 (8.8%); and HIV-associated dementia: 1 (1.3%) patients.

Table 2 shows the prevalence of mental health conditions in the study population. The most frequent disorders were: anxiety (21.3%); bipolar (11.3%); and substance dependence (8.8%). A total of 37.5% (26.3–48.7%) of the patients evaluated had at least one mental health condition.

### 3.3. Association between Sociodemographic, Clinical-HIV Variables and Mental Health Conditions with NCI Diagnosis

In the multivariate analysis, only time since HIV diagnosis and at least one UMHC showed a significant association with NCI diagnosis (Table 3).

### 3.4. Relation of Mental Health Conditions Score and NCI

Regarding differences in the score of each mental health conditions depending on NCI diagnosis, observed were significant differences in anxiety (*p* = 0.028), alcohol dependence (*p* = 0.029) and post-traumatic stress (*p* = 0.039). For all cases, PLWHIV with NCI diagnosis showed higher levels of symptoms of these mental health conditions in comparison to their counterparts without NCI diagnosis (Table 4).

## 4. Discussion

Based on the results obtained in this study, it would be necessary to establish specific protocols to diagnose mental health conditions in PLWHIV. We propose that an exhaustive mental health conditions evaluation through a previously validated questionnaire, such as the Millon Clinical Multiaxial Inventory-III, should be performed, at least once a year, in PLWHIV followed up in HIV infection units. Further, this exhaustive evaluation should be always done before an NCI evaluation. In case of a positive result, the patient should be referred to a mental health unit.

Results of the present study show a high prevalence of UMHC in PLWHIV without comorbidities with good adherence to ART and long-term suppressed viremia. This prevalence was found even though there was previous exclusion of those participants with known mental health conditions after reviewing electronic clinical records and performing the HADS questionnaire to discard anxiety or depression symptoms in “healthy” PLWHIV. Surprisingly, after the evaluation with the MCMI-III questionnaire, it was found a high proportion of mental health conditions in the analyzed sample, including depression, anxiety, bipolar and substance abuse, among others, non-previously identified through the employment of classical used screening questionnaires, such as HADS. Although substance abuse was an exclusion criterion, no previously self-reported drugs and alcohol dependence was identified in 8.8% and 2.5% of patients, respectively. In summary, 37.5% of the patients presented at least one mental health condition, being anxiety and bipolar disorders the most prevalent.

As it has been reported previously, the prevalence of NCI is high (26.3%) in the studied population [11]. In the multivariate analysis, more than ten years since HIV diagnosis (ORa 5.0 (1.2–21.6)) and having at least one mental health condition (ORa 6.3 (1.9–20.8)) were associated with NCI diagnosis. Higher scores in anxiety, alcohol dependence and post-traumatic stress mean were observed in patients with NCI diagnosis.

The results of this research are in accordance with previous literature, in which it has been demonstrated that there is a high prevalence of mental health conditions in different HIV populations. Patients with HIV infection can live traumatic life events [36], are exposed to side effects of antiretroviral drugs and suffer non-AIDS comorbidities which can influence on health behaviors [37]. Major depression, dysthymia and anxiety disorders are frequent in PLWHIV and more prevalent in comparison to the general population without HIV infection when groups are matched by age and sex [17]. UMHC could have severe negative consequences, for instance, depression has been associated to less adherence to ART resulting in virological failure, decline in lymphocyte CD4+ recount [38], resistance to antiretroviral drugs and increase in mortality [39,40]. Other authors have associated depression with risky sexual practices for HIV transmission, such as promiscuity, anonymous sex and sex relations without the use of condoms [41,42].

A higher percentage of HIV people report abuse of toxic substances and illegal drugs compared with the general population [43]. Chronic alcohol consumption is associated to lower adherence to ART [44], caloric-protein malnutrition, immunosuppression, virological failure [45], chronic liver disease [46] and alteration of antiretroviral drugs metabolism [47]. In this research, we observed a minor proportion of patients who are dependent on addictive substances, something that was unknown to HIV clinicians and was not self-reported previously by patients. Chemsex-related drug use is an escalating public health issue among men who have sex with men, associated with significant physical, biomedical and psychosocial harm [48]. Generally, Chemsex is not self-reported by the patients to HIV clinicians and it is not identified as drug abuse by patients themselves. Long-term outcome of HIV patients with undiagnosed substance abuse is unknown.

Although we excluded patients with symptomatology of anxiety and depression using the HADS questionnaire and reviewing clinical records, our results show that there are still UMHC not identified by these evaluation strategies which could influence in the diagnosis of NCI. Based on previous research, there is some controversy in this regard. Fialho explored the relationship between depressive symptoms and neuropsychological performance in women living with HIV, some of them with bad adherence to ART and detectable HIV-VL. In the multivariate model, only depressive symptoms were associated with NCI [29]. Further, the studies from the CHARTER group (CNS HIV Antiretroviral Effects Research) and other groups have demonstrated an association between mental health conditions and NCI [9,31,49,50]. However, Cysique affirms that depression and anxiety do not affect NCI [30]. Authors of the PIVOT study [15] and others [23,27] describe factors associated with NCI in PLWHIV on stable combination ART, a population quite similar to our study, not reporting association between depression and NCI. In these studies, depression and anxiety were the only mental health conditions analyzed, being evaluated by screening questionnaires specifically designed for the identification of symptomatology, but not as mental health conditions properly [23,27]. Other mental health conditions that have demonstrated to have a significant impact on cognitive functioning, such as bipolar disorder [32,51], drug or alcohol abuse [32,52] or delusional disorder [53], among others, were not evaluated in these studies. The poor assessment of mental health conditions and the use of screening methods may be the cause of the lack of congruence in the literature on the relationship between mental health conditions and NCI. The obtained results point out that probably screening tests might not be adequate to discard mental health conditions in PLWHIV.

Based on all of this information mentioned above and with the aim to identify which specific mental health conditions could be related to NCI diagnosis, differences between groups with or without NCI diagnosis were conducted. As it has been described by other authors, higher scores in anxiety, alcohol dependence and post-traumatic stress in PLWHIV with NCI diagnosis were, as in [24,32,54].

We highlight the high prevalence of NCI in well-controlled PLWHIV, without comorbidities including mental health conditions. Therefore, pathogenic mechanisms such as HIV neurotropism or chronic inflammation could play a main role in NCI development in this selected population [11].

Although the present study entails an advance in the comprehension of the association between mental health conditions and NCI in PLWHIV, some limitations should be taken into account. The design of the study only allows to measure the association of mental health conditions with NCI, avoiding establishing any causality. Further, the inclusion and exclusion criteria used in our study do not permit extrapolating the results to the whole population of PLWHIV. However, our study has several methodological strengths compared to other studies and this allows us to draw significant conclusions. In comparison to previous research, we recruited a population not enough studied in neuropsychological research, that is to say, only well-controlled PLWHIV without comorbidities. Secondly, we have done an exhaustive and comprehensive analysis of the main mental health conditions. Moreover, to our knowledge, the Millon Clinical Multiaxial Inventory-III questionnaire has not been previously employed to discard the effects of mental health conditions on NCI diagnosis in PLWHIV.

## 5. Conclusions

In conclusion, our study highlights that UMHC are frequent in PLWHIV, even in well-controlled HIV subjects. These disorders are not recognized by clinicians, not self-reported by patients or not identified by basic screening questionnaires. Some of these UMHC could have serious implications in public health, affect the control of HIV infection and even act as confounders of NCI diagnosis.

Basic screening instruments such as HADS are not enough to exclude mental health conditions in PLWHIV. Other mental health conditions than anxiety and depression should be evaluated in this population, for instance, in patients with bad ART adherence, frequent virologic failure or before NCI diagnosis. These tests could avoid NCI over diagnosis and avoiding the added stress of a misdiagnosis.

Based on the results, it is necessary to carry out more neuropsychological studies in PLWHIV given the vulnerability of this population using diagnostic questionnaires to evaluate mental health conditions. Future studies should develop longitudinal studies in order to identify how mental health conditions have an effect on NCI and how they could be detrimental to cognitive functioning, analyzing their particular influence on several cognitive domains.

## Figures and Tables

**Table 1:** Descriptive analysis of people living with HIV (PLWHIV) included in the study (*N* = 80).

Sociodemographic and Clinical Characteristics			Variables
Male, *n* (%)			77.5 (62)
Age (years), mean ± SD			46.1 ± 7.6
Age (≥44 years), *n* (%)			56.3 (45)
Educational levels, *n* (%):			
	Primary or no education		33.8 (27)
	Secondary education		41.3 (33)
	University education		25.0 (20)
Clinical HIV variables			
HIV transmission mechanism, *n* (%)			
	Former-IDU ^1^		7.5 (6)
	Sexual:		92.4 (74)
		MSM ^2^	58.8 (47)
		HTX ^3^	33.8 (27)
Time since HIV diagnosis (years), mean ± SD			12.4 ± 7.1
≥10 years since HIV diagnosis, *n* (%)			61.3 (49)
Current CD4+ lymphocytes (cells/µL.), mean ± SD			653.5 ± 219.5
Current CD4+ count < 500 cells/µL., *n* (%)			23.8 (19)
Nadir CD4+ lymphocyte (cells/µL.), mean ± SD			246.0 ± 151.6
Nadir CD4+ count ≤ 350 cells/µL, *n* (%)			76.3 (61)

^1^ Previous intravenous drug users; ^2^ men who have sex with men; ^3^ heterosexual.

**Table 2:** Prevalence of mental health conditions according to the Millon Clinical Multiaxial Inventory-III (*N* = 80).

Mental Health Conditions	Prevalence (95% CI) ^1^	*n*
Anxiety	21.3 (11.7–30.8)	17
Somatoform	5.0 (1.4–12.3)	4
Bipolar	11.3 (3.7–18.8)	9
Dysthymia	2.5 (0.3–8.7)	2
Alcohol dependence	2.5 (0.3–8.7)	2
Drug dependence	8.8 (1.9–15.6)	7
Post-traumatic stress	-	0
Thought disorder	5.0 (1.4–12.3)	4
Major depression	-	0
Delusional disorder	6.3 (2.0–14.0)	5
At least one mental health condition	37.5 (26.3–48.7)	30

^1^ Confidence interval 95%.

**Table 3:** Association between sociodemographic, clinical HIV variables and mental health conditions with neurocognitive impairment (NCI) diagnosis (*N* = 80).

Sociodemographic, Clinical HIV Variables and Mental Health Conditions		Neurocognitive Impairment		Orc ^3^ (95% CI) ^4^	*p*_c_^5^	Ora ^6^ (95% CI) ^4^	*p*_a_^7^
		%	(*n*/*N*)				
Gender							
	Male	22.6	(14/62)	0.5 (0.2–1.5)	0.224	-	-
	Female	38.9	(7/18)	1			
Age							
	≥44 years	31.1	(14/45)	1.8 (0.6–5.1)	0.263	-	-
	<44 years	20.0	(7/35)	1			
Education level							
	Non/Primary	37.0	(10/27)	2.7 (1.0–7.0)	0.118	-	-
Secondary/University		20.8	(11/53)	1			
HIV mechanism transmission							
	Former-IDU ^1^	33.3	(2/6)	1.5 (0.3–8.6)	0.650	-	-
	Sexual ^2^	25.7	(21/80)	1			
Time since diagnosis							
	≥10 years	36.7	(18/49)	5.5 (1.4–20.4)	0.007	5.0 (1.2–21.6)	0.030
	<10 years	9.7	(3/31)	1			
Current CD4+ (cells/µL)							
	<500/µL	21.1	(4/19)	0.7 (0.2–2.4)	0.767	-	-
	≥500/µL	27.9	(17/61)	1			
Nadir CD4+ (cells/µL)							
	≤350/µL	32.8	(20/61)	8.8 (1.1–70.5)	0.018	6.6 (0.7–59.4)	0.095
	>350/µL	5.3	(1/19)	1			
At least one mental health condition							
	Presence	46.7	(14/30)	5.4 (1.8–15.7)	0.001	6.3 (1.9–20.8)	0.002
	Absence	14.0	(7/50)	1			

^1^ Previous intravenous drug users; ^2^ sexual transmission; ^3^ crude odds ratio; ^4^ confidence interval 95%; ^5^ crude statistical significance; ^6^ adjusted odds ratio; ^7^ adjusted statistical significance.

**Table 4:** Results of comparing the means for NCI or not samples in mental health conditions (*N* = 80).

Mental Health Conditions	Neurocognitive Impairment				*t ^3^*	*p ^4^*	*d ^5^*
	Yes (*n* = 21)		No (*n* = 59)				
	M ^1^	SD ^2^	M ^1^	SD ^2^			
Anxiety	50.9	32.6	33.1	30.8	2.2	0.028	0.56
Somatoform	34.8	27.7	27.4	26.5	1.1	0.285	0.28
Bipolar	54.5	22.9	43.4	24.4	1.8	0.072	0.47
Dysthymia	31.2	25.3	20.4	24.1	1.8	0.085	0.44
Alcohol dependence	44.6	25.3	31.4	22.8	2.2	0.029	0.55
Drug dependence	41.0	27.9	32.9	25.2	1.2	0.222	0.30
Post-traumatic stress	32.3	22.2	20.2	22.9	2.1	0.039	0.53
Thought disorder	34.3	28.7	26.4	24.6	1.2	0.229	0.30
Major depression	25.7	28.6	21.7	23.0	0.6	0.569	0.15
Delusional disorder	37.2	34.5	33.1	31.6	0.5	0.618	0.12

^1^ Media; ^2^ standard deviation; ^3^ *t*-test for comparing two means; ^4^ *p*: statistical significance; ^5^ *d*: Cohen’s d.
